# Analysis on in vitro effect of lithium on telomere length in lymphoblastoid cell lines from bipolar disorder patients with different clinical response to long-term lithium treatment

**DOI:** 10.1186/s40246-022-00418-8

**Published:** 2022-10-17

**Authors:** Alessio Squassina, Anna Meloni, Donatella Congiu, Panagiotis Bosganas, George P. Patrinos, Rixing Lin, Gustavo Turecki, Giovanni Severino, Raffaella Ardau, Caterina Chillotti, Claudia Pisanu

**Affiliations:** 1 Laboratory of Pharmacogenomics, Department of Biomedical Sciences, Section of Neuroscience and Clinical Pharmacology, University of Cagliari, Sp 8 Sestu-Monserrato, Km 0.700, Mosnerrato, 09042 Cagliari, Italy; 2 Laboratory of Pharmacogenomics and Individualized Therapy, School of Health Sciences, Department of Pharmacy, University of Patras, Patras, Greece; 3 College of Medicine and Health Sciences, Department of Genetics and Genomics, United Arab Emirates University, Al-Ain, Abu Dhabi UAE; 4 Zayed Center for Health Sciences, United Arab Emirates University, Al-Ain, Abu Dhabi UAE; 5 McGill Group for Suicide Studies, Department of Psychiatry, Douglas Mental Health University Institute, McGill University, Montreal, QC Canada; 6 Integrated Program in Neuroscience, McGill University, Montreal, QC Canada; 7 Unit of Clinical Pharmacology, University Hospital Agency of Cagliari, Cagliari, Italy

**Keywords:** Bipolar disorder, Mood disorders, Accelerated aging, Cellular systems, Neural precursors, Lymphoblastoid cell liens, Lithium, In vitro, Aging, Telomeres

## Abstract

**Background:**

It has been suggested that bipolar disorder (BD) is associated with clinical and biological features of accelerated aging. In our previous studies, we showed that long-term lithium treatment was correlated with longer leukocyte telomere length (LTL) in BD patients. A recent study explored the role of TL in BD using patients-derived lymphoblastoid cell lines (LCLs), showing that baseline TL was shorter in BD compared to controls and that lithium in vitro increased TL but only in BD. Here, we used the same cell system (LCLs) to explore if a 7-day treatment protocol with lithium chloride (LiCl) 1 mM was able to highlight differences in TL between BD patients clinically responders (Li-R; *n* = 15) or non-responders (Li-NR; *n* = 15) to lithium, and if BD differed from non-psychiatric controls (HC; *n* = 15).

**Results:**

There was no difference in TL between BD patients and HC. Moreover, LiCl did not influence TL in the overall sample, and there was no difference between diagnostic or clinical response groups. Likewise, LiCl did not affect TL in neural precursor cells from healthy donors.

**Conclusions:**

Our findings suggest that a 7-day lithium treatment protocol and the use of LCLs might not represent a suitable approach to deepen our understanding on the role of altered telomere dynamics in BD as previously suggested by studies in vivo.

**Supplementary Information:**

The online version contains supplementary material available at 10.1186/s40246-022-00418-8.

## Background

Bipolar disorder (BD) is a severe, chronic, psychiatric disorder characterized by recurrent episodes of depression and mania/hypomania alternating with intervals of well-being. It affects 0.8–1.2% of the general population [1] and is associated with high disability and premature mortality [2]. Like other severe mental disorders, the life expectancy for BD patients is reduced up to 10–20 years compared to the general population [3]. This is mainly contributed by a higher prevalence of comorbid chronic disorders compared to individuals without mental illnesses, and especially by age-related medical conditions with an inflammatory component, such as cardiovascular and metabolic disorders [4]. This evidence has led to the hypothesis that accelerated aging and inflammation may play a central role in the etiopathogenesis of mental disorders [5]. Several studies reported altered markers of aging in BD patients, including increased epigenetic and brain age, as well as shorter telomere length [6–8]. Considering the well-known neuroprotective effect of lithium [9], its anti-aging properties have been largely studied [10] with evidence suggesting that lithium reduces epigenetic aging (an effect shared with other mood stabilizers [11]) and protects telomeres from shortening [12–14]. Recently, we showed that leukocyte telomere length (LTL) was positively correlated with duration of lithium treatment in BD patients [13, 14] and that those exposed to lithium for at least one year had longer LTL compared to patients never treated with lithium [14]. Even though these findings support the hypothesis that lithium might counteract accelerated telomere shortening, whether this effect plays a role in modulating lithium’s clinical efficacy remains to be clarified. Indeed, results on this matter have been so far incongruent, with some studies reporting longer telomeres in BD patients responders to long-term lithium treatment [15] and others reporting no differences [13]. This lack of replicability might be accounted for by several factors typically affecting clinical studies on psychiatric disorders, with the limited access to patients in monotherapy with lithium being likely one of the major contributors. To this regard, the use of cellular systems generated from BD patients could be a promising tool to explore the effect of pharmacological treatments. Indeed, cell lines can be grown under controlled conditions and treated with lithium in monotherapy. Based on these assumptions, in a recent study Fries and coworkers [16] showed that TL was shorter in lymphoblastoid cell lines (LCLs) from BD patients compared to controls and that lithium treatment in vitro significantly increased TL in patients, but not in controls. This finding supports the notion that LCLs could represent a reliable tool to investigate telomere dynamics in BD. However, to date the study from Fries and colleagues is the only published work with this study design, and as such more efforts are needed to explore the utility of LCLs in this field. Moreover, the study by Fries et al. did not explore if lithium in vitro had differential effect on TL in LCLs from responders or non-responders to prophylactic lithium treatment.

Here, we present the findings from a study where we sought to further elucidate the effect of lithium on TL and to explore whether this effect could be correlated with lithium’s clinical efficacy. To this aim, LCLs from BD patients responders or non-responders to long-term lithium treatment and controls were grown with or without lithium chloride (LiCl) 1 mM for 7 days and TL was measured.

## Results

One Li-NR was excluded from the study since TL for this individual was a significant outlier based on the Grubb’s test. As such, the studied sample included 15 Li-R, 14 Li-NR and 15 HC. Li-R and Li-NR significantly differed for the number of episodes and history of suicide attempts (Table 1). Therefore, all the tests comparing Li-R and Li-NR were controlled for these variables.

**Table 1:** Demographic and clinical characteristics of the studied sample

Variables	Li-R (*n* = 15)	Li-NR (*n* = 14)	*p* (Li-R vs. Li-NR)	BD (*n* = 29)	HC (*n* = 15)	*p* (BD vs HC)
Age (mean ± SD)	42.7 ± 15.6	42.9 ± 12.9	0.97	42.8 ± 14.1	42.4 ± 5.1	0.89
Sex (M/F)	7/8	7/7	1	14/15	7/8	1
Age at onset (mean ± SD)	27.7 ± 13.7	25.1 ± 7.9	0.54		/	
Years of illness (mean ± SD)	14.7 ± 7.4	13.3 ± 9.9	0.66		/	
Years of lithium (mean ± SD)	7.4 ± 7.7	10.4 ± 5.1	0.24		/	
TS of the Alda scale (mean ± SD)	8.6 ± 0.91	0.00 ± 0.00	/		/	
Number of episodes (mean ± SD)	6.5 ± 4.5	13.3 ± 5.9	**0.002**		/	
Psychotic symptoms (Y/N)	6/9	11/3	0.06*		/	
Suicide attempts (Y/N)	1/14	6/8	0.03*		/	

*M* male; *F* female; *SD* standard deviation; *Y* yes; *N* no; *TS* total score; *Li-R* lithium responders; *Li-NR* lithium non-responders; *BD* bipolar disorder; *HC* non-psychiatric controls; and *p:* *p* value of the test

*p value of the Fisher exact test. *P* values in bold are statistically significant (*p* < 0.05)

TL was not correlated with age or with any of the demographic or clinical variables tested.

**Fig. 1:**
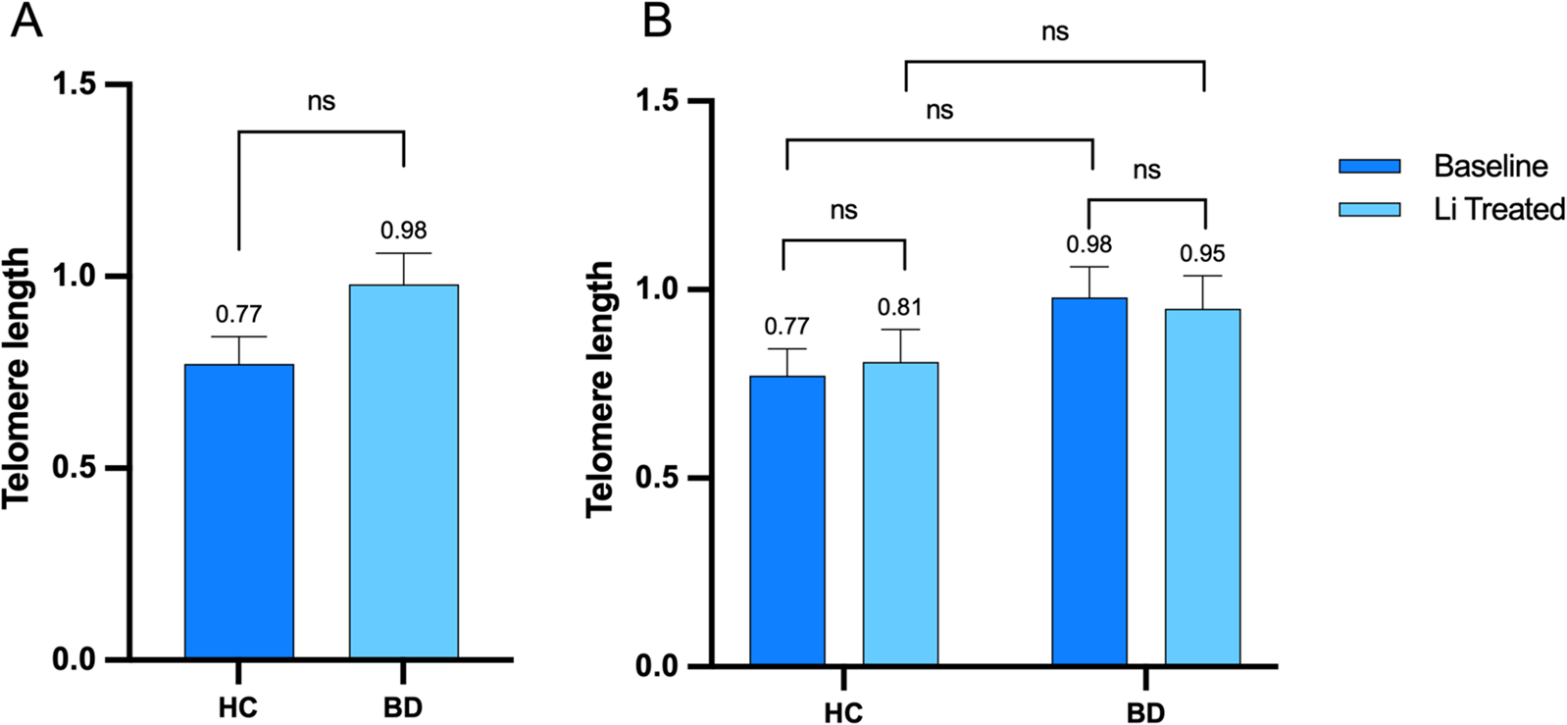
Comparison of telomere length in bipolar disorder patients and non-psychiatric controls. **A**. Comparison of baseline telomere length between bipolar disorder and non-psychiatric controls. **B**. Comparison of the effect of in vitro lithium treatment in bipolar disorder patients and non-psychiatric controls. *HC* Non-psychiatric controls; *BD* bipolar disorder; ns not statistically significant

BD patients had longer TL compared to HC, though the difference did not reach statistical significance (model controlled for age: *F* = 2.05, *p* = 0.124, Fig. 1A). As suggested by our previous work [14, 17], this finding could be determined by the fact that all patients had been under lithium treatment for several years when recruited for setting LCLs. There was no effect of LiCl on TL in either of the diagnostic groups, and there was no difference between BD and HC (statistics for treatment*diagnosis interaction, *F* = 2.07; *p* = 0.18, Fig. 1B).

**Fig. 2:**
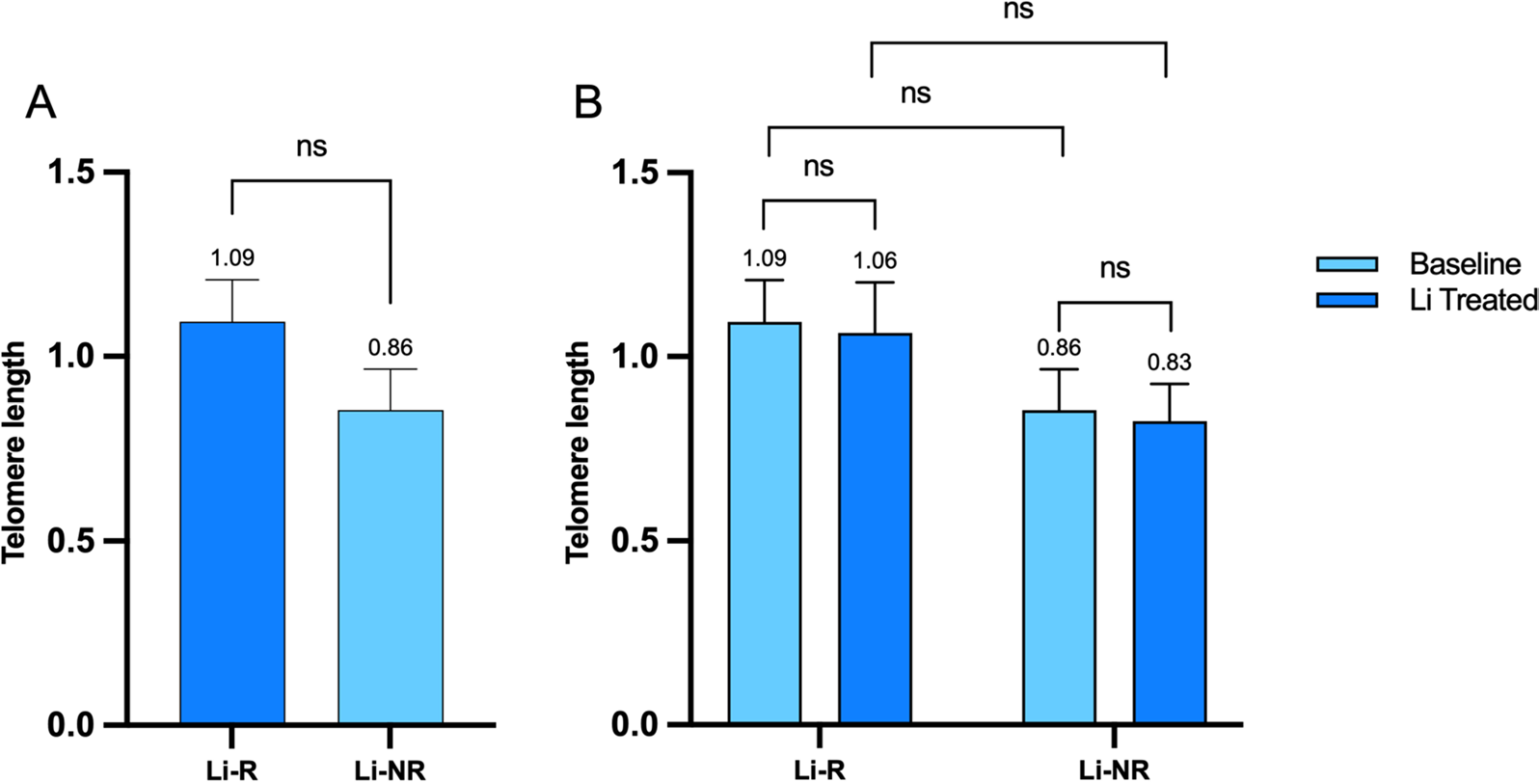
Comparison of telomere length in bipolar disorder patients responders or non-responders to long-term lithium treatment. **A**. Comparison of baseline telomere length between bipolar disorder patients responders or non-responders to long-term lithium treatment. **B**. Comparison of the effect of in vitro lithium treatment on telomere length between bipolar disorder patients responders or non-responders to long-term lithium treatment. *Li-R* Lithium responders; *Li-NR* lithium non-responders; *ns* not statistically significant

TL at baseline was not significantly different in Li-R compared to Li-NR in a model controlled for age, number of episodes and suicide attempts (corrected model *F* = 1.20, *p* = 0.39, Fig. 2A). Moreover, there was no significant difference in the effect of LiCl on TL between Li-R and Li-NR in the repeated measures model (effect of treatment*response group: *F* = 0.00, *p* = 1.00, Fig. 2B). In order to explore if lithium influenced TL in a neural cell line (neurons are the target of lithium), we grown human-derived neural precursors (NPCs) with or without LiCl for 7 days and showed no effect of treatment on TL (Additional file 1: Fig. S1).

## Discussion

Previous studies support the hypothesis that BD could be associated with clinical and biological markers of accelerated aging, including telomere shortening [18]. Nevertheless, findings on TL in BD remain controversial, and the question whether BD patients present shorter or longer telomeres compared to controls remains unanswered [19]. On the other hand, there is robust evidence suggesting that lithium exposure is associated with longer telomeres [13–15, 20, 21]. This effect could be modulated by lithium’s effect on several genes involved in telomere dynamics [20], with a major role played by the human telomerase reverse transcriptase gene (hTERT). Indeed, several studies showed that lithium treatment is associated with increased expression of hTERT both in animals [22, 23] and humans [13, 22, 23]. Overall, findings suggest that the modulation of telomere dynamics could be one of the mechanisms through which lithium may explicate its anti-aging properties, but do not allow understanding if elomeres play a role in response to lithium's mood stabilizing properties. In 2013, Martinsson and colleagues [15] showed for the first time that patients responding to long-term lithium treatment had longer telomeres compared to non-responders, but this finding was not replicated in a further independent investigation [13]. The paucity of studies in this field significantly limits our capability to disentangle the complex matter of the interplay between telomeres and lithium’ efficacy, calling for more action. The main difficulties encountered when carrying out clinical studies with this aim are represented by the limited access to BD patients treated with lithium in monotherapy and by the large number of confounding factors potentially impacting on TL which are often difficult to account for in the analytical models. One way to partly overcome these limits is the use of cellular models, such as patients-derived neurons or peripheral cells lines. Indeed, cell systems allow to control for several potential confounding factors impacting on clinical studies, since they are grown under controlled conditions and can be treated in monotherapy with the needed medications. In the case of lithium, this approach remains still limited in that chronic lithium treatment (intended as prophylactic treatment) cannot be mimicked in vitro. Moreover, patients-derived neurons or peripheral cells do not necessarily allow exploring brain dysfunctions. Nevertheless, in previous studies we showed that the use of peripheral cell lines (such as LCLs) to study psychiatric disorders could be informative and correlated with brain abnormalities, especially when utilized to explore the effect of perturbating features, such as drug exposure [24–27]. Recently, Fries and colleagues [16] used LCLs derived from 14 BD patients and 14 controls to explore the effect of in vitro lithium treatment on TL and epigenetic aging. Findings showed that, even in LCLs, TL was correlated with chronological age and that was shorter in BD compared to HC. Interestingly, lithium treatment for 7 days increased TL but only in BD and not in controls suggesting that lithium might interfere with TL only when needed.

In our current study, we applied a similar study design but extended the analysis to two groups of BD patients with different clinical responses to lithium to identify possible lithium-induced differences in TL between response groups. Our results did not confirm the findings from Fries et al. [16] and show that Li-R and Li-NR do not differ for either TL or the effect of exposure to in vitro lithium treatment.

The lack of replication of the findings from Fries et al. could be determined by several factors which, at this stage, can only be hypothesized. Firstly, while similar, the sample size of Fries and our study could be underpowered to detect robust differences among studied groups. We observed longer LT in BD compared to HC as well as in Li-R compared to Li-NR, but the difference was not statistically significant, and this could be determined by the small number of patients included. Our results do not replicate previous in vivo investigations either, but important limitations and differences should be considered when interpreting findings. Indeed, in vivo studies showed correlation between TL and duration of lithium treatment in chronically treated patients, sometimes treated for several years if not decades, a condition that is impossible to mimic in vivo. Lastly, information on comorbidities with medical disorders or duration of treatment with other psychiatric medications other than lithium was not available for our patients, and, as such, we cannot exclude a confounding effect of these variables on our findings.

## Conclusions

In conclusion, our study suggests that the use of LCLs and a 7-day protocol of lithium treatment in vitro could not be appropriate to explore the role of telomere dynamics in modulating clinical efficacy of lithium as a mood stabilizer. Indeed, regulation of telomere dynamics is a finely regulated process, the mechanisms involved are complex and to be properly addressed they would likely require different study designs implementing long periods of drug exposure and observation, and possibly sampling at multiple timepoints in a longitudinal setting.

Thus, further studies on larger datasets and different cellular systems (such as patients-derived neurons) might be needed to help better disentangling the complex interplay between telomeres and lithium’s clinical efficacy.

## Methods

### Sample

The sample of patients and controls was recruited at the Lithium Clinic of the Clinical Psychopharmacology Centre of the University Hospital of Cagliari, Italy. The lithium clinic is a specialized unit in the management of BD patients and has been active for more than 40 years, being also involved in several research initiatives aimed at exploring the molecular underpinnings of response to mood stabilizers. The current study included a sample of 30 BD patients retrospectively evaluated for lithium response and selected from an existing cohort of 150 patients recruited in the last 20 years and for whom LCLs were stored in our laboratory. The study sample was selected based on the following criteria: LCLs available in our biobank; fulfilling the inclusion criteria to be characterize for lithium response with the Alda scale (which requires lithium monotherapy for at least one year); lithium response score at the two extreme ends of the Alda scale (as described later in the methods section); and age and sex matched.

Similarly, 15 age- and sex-matched non-psychiatric controls (HC) were selected from a cohort of 100 individuals without any personal or family history of psychiatric conditions based on self-report. Patients were diagnosed according to Research Diagnostic Criteria (RDC) [28] and DSM-IV criteria, using personal semi-structured interviews (Schedule for Affective Disorder and Schizophrenia Lifetime Version (SADS-L)) [29] and a systematic review of their medical records. Exclusion criteria included comorbidities with any disorder of the DSM-IV axes I and II. Clinical response to maintenance treatment with lithium was evaluated using the “Retrospective Criteria of Long-Term Treatment Response in Research Subjects with Bipolar Disorder” (Alda scale) [30, 31], as previously described [32]. Briefly, the scale measures the degree of improvement in the course of treatment (score A) weighted against a number of clinical factors considered relevant in determining whether or not the improvement observed is due to lithium treatment (score B). The degree of response for each patient is quantified with a score from 0 to 10 (total score), obtained by subtracting the score B from the score A. Patients with total score (TS) equal to 7 or higher are considered responders (R) to treatment. For this study, we selected patients at the two extreme ends of the TS: 15 lithium responders (Li-R; total score > 7) and 15 non-responders (Li-NR, total score = 0). Demographic and clinical characteristics of patients and controls are reported in Table 1.

The research protocol followed the principles of the Declaration of Helsinki and was approved by the Ethics Committee of the University of Cagliari, Italy (approval number: 348/FC/2013, approved in date: 21 June 2013). All participants signed informed written consent after a detailed description of the study procedures.

### Lymphoblastoid cell lines (LCLs)

LCLs were already available for the entire cohort and were generated following standard procedures. Briefly, Epstein–Barr virus immortalized LCLs were established from lymphocytes and stored in liquid nitrogen at the time of enrollment in previous molecular studies [25]. For the present study, LCLs from selected patients (and with passage numbers < 5) were thawed and cultured using a standard protocol previously described [33] Once LCLs reached the required cell count (6–9 × 10^6^ cells), two equivalent aliquots were transferred into separate flasks. One aliquot was cultured with medium supplemented with 1 mM lithium chloride (LiCl), while the other one was cultured with drug-free medium, under identical conditions. After 7 days of treatment, cells were harvested for DNA isolation.

### Neural precursors cells (NPCs)

Aiming to test the effect of lithium on TL also in a neural cell line, we grown human NPCs (ReNcell; Sigma-Aldrich) with or without LiCl 1 mM for 7 days. Briefly, NPCs were maintained in STEMdiff neural progenitor medium (STEMCELL Technologies) supplemented with 1 mM LiCl (sigma-Aldrich) (*N* = 6) or PBS vehicle control (*N* = 6) for one week before harvesting. NPCs were grown in a 5% CO2 humidified incubator at 37 °C, and culture media were changed every 48 h. NPCs were tested for mycoplasma contamination and authenticated by their manufacturer.

### DNA extraction

DNA was extracted from LCLs and NPCs using the salting-out method [34]. DNA quantification and quality evaluation were performed through spectrophotometric analysis (NanoDrop 2000, Thermo Scientific, Waltham, MA, USA).

### Measurements of leukocyte telomere length with quantitative PCR

Relative LTL was assessed according to the quantitative PCR (qPCR) method as previously described [35]. Samples were processed in triplicates both for the telomere (Tel) and for the single-copy gene (hemoglobin-b, Hgb) using Platinum® SYBR® Green qPCR SuperMix-UDG w/ROX (Thermo Fisher Scientific, Waltham, MA, USA) on a StepOnePlus TM Real-Time PCR System (Thermo Fisher Scientific). Primer sequences were as follows: Tel-1, 50-GGTTTTTGAGGGTGAGGGTGAGGGTGAGGGTGAGGGT-30, Tel-2, 50-TCCCGACTATCCCTATCCCTATCCCTATCCCTATCCCTA-30; Hgb1, 50-GCTTCT-GACACAACTGTGTTCACTAGC-30, Hgb2, 50-CACCAACTTCATCCACGTTCACC-30. The PCR temperature conditions were 95 for 15 s and 60 °C for 1 min for Hgb. Specificity was assessed through the dissociation curve included in each plate. A pool of control samples was included in each plate as a calibrator, and LTL was calculated using the 2 ^−DDCT^ method where DDCT = DCT sample–DCT calibrator and DCT sample = CT Tel–CT Hgb.

### Statistical analysis

Normality of the distribution was assessed using the Shapiro–Wilk test. Grubb’s test was used to identify outliers. The association between LTL and quantitative or categorical variables was assessed using partial correlation test controlled for age or Mann–Whitney’s U test, respectively. Differences in baseline TL between diagnostic (BD versus HC) or lithium response groups (Li-R versus Li-NR) were analyzed using general linear models controlled for age and other variables showing significant difference between the studied groups, as described in the results section. The difference in the effect of in vitro lithium treatment based on diagnosis or clinical response to lithium was tested with general linear models for repeated measures controlled for the variables as described above. The significance threshold was set at *p* < 0.05. Analyses were conducted using GraphPad Prism v. 9 (GraphPad Software, CA USA) and SPSS v. 27 (IBM Corporation, NY, USA).

## Supplementary Information

**Additional file 1.** **Fig.** **S1** Effect of lithium chloride 1mM for 7 days on telomere length in human-derived neural precursors cells (NPCs). Li: lithium treated; ns: not significant.

## Data Availability

The datasets used and/or analyzed during the current study are available from the corresponding author on reasonable request.

## Abbreviations

BD: Bipolar disorder
HC: Non-psychiatric controls
TL: Telomere length
Hgb: Hemoglobin-b
LCL: Lymphoblastoid cell lines
Li-R: Lithium responders
Li-NR: Lithium non-responders
LTL: Leukocyte telomere length
LiCl: Lithium chloride
mM: Millimolar
hTERT: Human telomerase reverse transcriptase gene
