# Supplementary figures and images for: Analysis on in vitro effect of lithium on telomere length in lymphoblastoid cell lines from bipolar disorder patients with different clinical response to long-term lithium treatment
Source: Hum Genomics. 2022 Oct 17;16:45. doi: 10.1186/s40246-022-00418-8 (PMC9575289; doi:10.1186/s40246-022-00418-8)

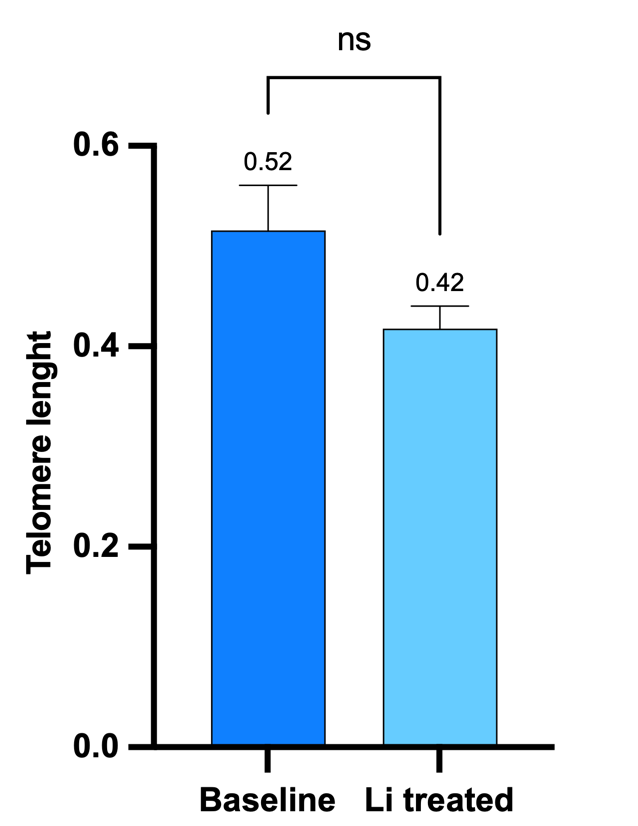

Supplement: Supplementary file 1 — Additional file 1. Fig. S1 Effect of lithium chloride 1mM for 7 days on telomere length in human-derived neural precursors cells (NPCs). Li: lithium treated; ns: not significant. [file 40246_2022_418_MOESM1_ESM.tiff]
